# *BRAF^V600E^*-mutated ovarian serous borderline tumors are at relatively low risk for progression to serous carcinoma

**DOI:** 10.18632/oncotarget.27326

**Published:** 2019-12-03

**Authors:** M. Herman Chui, Susanne K. Kjaer, Kirsten Frederiksen, Charlotte G. Hannibal, Tian-Li Wang, Russell Vang, Ie-Ming Shih

**Affiliations:** ^1^ Department of Pathology, The Johns Hopkins University School of Medicine, Baltimore, MD, USA; ^2^ Department of Obstetrics & Gynecology, The Johns Hopkins University School of Medicine, Baltimore, MD, USA; ^3^ Unit of Virus, Lifestyle and Genes, Danish Cancer Society Research Center, Copenhagen, Denmark; ^4^ Gynecologic Clinic, Juliane Marie Centre, Copenhagen University Hospital, Copenhagen, Denmark

**Keywords:** ovarian serous borderline tumor, mutation, genotyping, risk prediction, serous carcinoma

## Abstract

Ovarian serous borderline tumor (SBT) is a known precursor of low-grade serous carcinoma. While most SBTs are cured surgically, some progress to carcinoma and a risk predictor for malignant relapse is needed to ensure vigilant follow-up and additional treatment. Activating mutations in *KRAS* or *BRAF* are present in around 60% of SBTs, but their relative impact on progression is unclear. We performed mutational analysis of *KRAS* and *BRAF* on 201 SBTs identified from a longitudinal cohort of SBTs after centralized pathology review. Compared to wildtype and *KRAS*-mutated SBTs, *BRAF*-mutated group of SBTs were less likely to exhibit micropapillary variant histology (p < 0.0001), were more frequently Stage I (p = 0.0023) and had a lower prevalence of associated endosalpingiosis (p = 0.0069). The histologic feature of diffuse presence of tumor cells with dense eosinophilic cytoplasm, while significantly associated with the *BRAF^V600E^* mutation (p < 0.0001), is 62% sensitive and 93% specific in identifying tumors with this mutation. After adjusting for age and stage, the risk of subsequent serous carcinoma was lower for SBTs harboring *BRAF* (HR 0.27, 95% CI 0.08–0.93), but not *KRAS* (HR 1.00, 95% CI 0.45–2.23) mutations, in comparison to wildtype SBTs. This study establishes the potential utility of mutation testing for guiding clinical management of ovarian SBT and underscores the importance of accurate morphologic distinction of micropapillary SBT from SBT with eosinophilic tumor cells, given their disparate prognostic implications.

## INTRODUCTION

Ovarian serous borderline tumor (SBT) is a low-grade epithelial ovarian neoplasm, typically diagnosed at early stage and associated with an excellent prognosis. Compared to other ovarian epithelial tumors, this disease affects younger reproductive-age women. As its name implies, SBT is not an entirely benign condition and is associated with increased risk for subsequent development of invasive low-grade serous carcinoma (LGSC), which occurs in 4-7% of patients [1-3]. The emergence of invasive LGSC is attributed to disease progression and markedly decreases overall survival [2].

Prior studies have established SBT as the immediate precursor of LGSC, and mutations of *KRAS* and *BRAF* are detected in nearly equal proportions of approximately one third of SBTs, respectively [4, 5]. As an enigmatic pathologic entity, positioned between benign and malignant disease, an unusual feature of SBT is the ability to disseminate and seed onto the peritoneum, forming so-called “implants,” which is considered advanced stage (i.e. Stage II or above; SBT follows conventional staging for ovarian cancer). We have previously demonstrated that the vast majority of peritoneal implants and their corresponding SBTs harbor identical *KRAS* or *BRAF* mutations, supporting a clonal relationship [6]. These implants are the likely source that give rise to subsequent serous carcinomas after primary resection.

Most clinicopathological studies of SBTs, to date, have several limitations: i) lack of population-based design where tumors have been uniformly classified by pathologists with subspecialty expertise; ii) most cases were from tertiary care centers, causing potential selection bias; iii) small sample sizes; and iv) lack of long-term follow-up data. In a meta-analysis of 245 studies reporting on approximately 18,000 patients, follow-up was available in less than 25% [7]. The mean follow-up was only 7.4 years, which is insufficient to understand the natural history of this relatively indolent disease.

We have previously reported on the clinicopa-thologic features of a nation-wide, Danish population-based cohort of women with SBT, with long-term follow-up data (median 15 years, up to 36 years). Clinicopathologic risk factors for subsequent development of serous carcinoma include bilateral ovarian involvement, ovarian surface involvement, advanced stage (defined by the presence of implants, with invasive implants in particular), post-surgical residual disease and micropapillary histology [8]. With accessibility to clinical outcome data and tissue materials in Denmark, we are positioned to address the critical question of whether *KRAS* and *BRAF* mutation status could further improve upon risk prediction for malignant progression.

## RESULTS

Due to the stratified sampling, there was a higher proportion of women with advanced stage (Stage II or higher) among the 201 SBTs genotyped, compared to the remaining women in the cohort [120/201 (60%) vs 20/824 (2%), p <0.0001].

The frequency distribution of mutations is, as follows: *BRAF,* n = 52, *KRAS*, n = 95 and wildtype for both genes, n = 54. Mutations in *KRAS* were predominantly codon 12 glycine to aspartate (G12D, 56/95, 59%) and glycine to valine (G12V, 31/95, 33%) single-nucleotide substitutions. Of note, 4 (2%) SBTs had mutations in both *BRAF* and *KRAS*. For these, tumor cells were enriched by laser-capture microdissection and digital droplet PCR (ddPCR) was repeated to obtain mutant allele frequencies (MAF) within the tumor cell population. In all 4 SBTs, *BRAF^V600E^* was the predominant mutation, with *KRAS* mutation occurring in a minor subpopulation (MAF for *BRAF^V600E^*: 56%, 51%, 53% and 56%, and for *KRAS* mutations: 14%, 10%, 11% and 4.5%, respectively). As such, these SBTs were categorized amongst the 52 tumors in the *BRAF*-mutated group.

**Figure 1:**
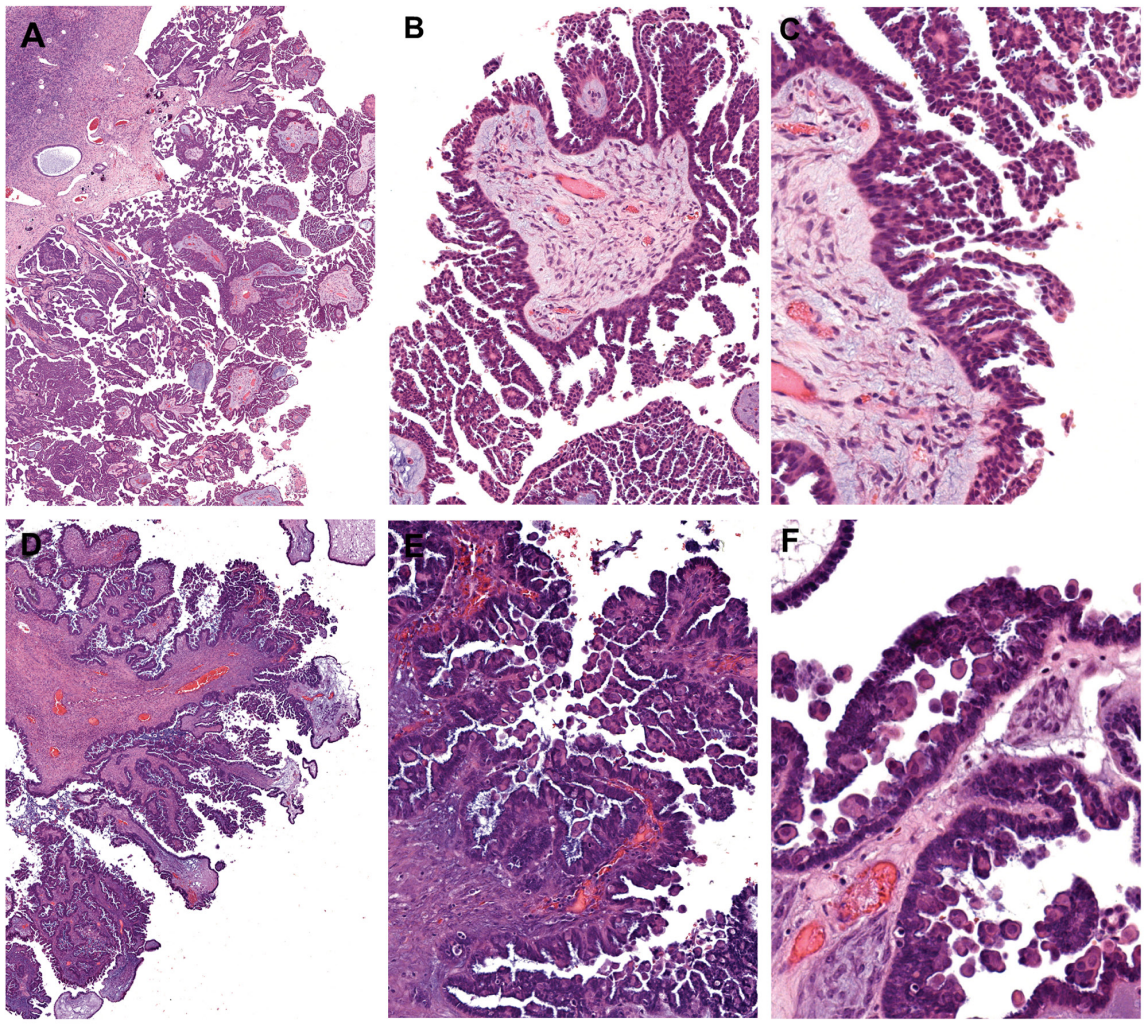
Histomorphologic features of micropapillary serous borderline tumor and *BRAF*-mutation-associated serous borderline tumor **(A-C)** Micropapillary serous borderline tumor demonstrating elongated slender papillae (5X greater in length than width) with non-hierarchical branching (“Medusa-head” pattern). **(D-F)** Serous borderline tumor with eosinophilic tumor cells, characteristic of the *BRAF^V600E^* mutation. At low-power magnification, the tumor has a crowded, hypercellular appearance, reminiscent of the micropapillary variant. However, closer inspection reveals short, blunt papillae with detached single cells and small cell clusters. These distinctive cells, which are often exfoliated, but also present within the tumor epithelium, exhibit dense/glassy eosinophilic cytoplasm occupying at least 50% the cell area, and at least twice the amount of cytoplasm compared with non-eosinophilic cells in the tumor. (A, D) 2X objective; (B, E) 10X objective; and (C, F) 20X objective.

Compared to wildtype and *KRAS*-mutated SBTs, *BRAF*-mutated group of SBTs were less likely to exhibit micropapillary variant histology (p < 0.0001, Figure 1A-1C) and more likely Stage I (p = 0.0023, Table 1). The prevalence of endosalpingiosis was also lower in women in *BRAF*-mutated SBTs (p = 0.0069).

**Table 1:** Clinicopathologic features of serous borderline tumors stratified by genotype

Feature	SBT genotype			*p* value
	*BRAF* mutation (n = 52)	*KRAS* mutation (n = 95)	Wildtype for *BRAF*/*KRAS* (n = 54)	
**Age, median (years/range)**	43 (20-72)	45 (16-88)	51 (15-96)	0.046
**Histologic type**				<0.0001
Typical (atypical proliferative serous tumor)	51 (98%)	87 (92%)	39 (72%)	
Micropapillary (non-invasive low-grade serous carcinoma)	1 (2%)	8 (8%)	15 (28%)	
**Laterality**				0.46
Unilateral	25 (48%)	36 (38%)	24 (44%)	
Bilateral	27 (52%)	59 (62%)	30 (56%)	
**FIGO Stage**				0.0023
I	30 (58%)	36 (38%)	14 (26%)	
>I	21 (40%)	59 (62%)	40 (74%)	
Unknown†	1 (2%)	0	0	
**Implant type (Stage >I only)**				0.20
Non-invasive	20/21	47/59	35/40	
Invasive	1/21	12/59	5/40	
**Microinvasion**				0.21
Present	6 (12%)	4 (4%)	3 (6%)	
Not identified	46 (88%)	91 (96%)	51 (94%)	
**Endosalpingiosis**				0.0069
Present	4 (8%)	26 (27%)	17 (31%)	
Not identified	48 (92%)	69 (73%)	37 (69%)	
**Capsule rupture**				0.20
Yes	12 (23%)	31 (33%)	12 (22%)	
No	35 (67%)	52 (55%)	37 (69%)	
Unknown†	5 (10%)	12 (13%)	5 (9%)	
**Surface involvement**				0.76
Yes	31 (60%)	61 (64%)	35 (65%)	
No	20 (38%)	30 (32%)	19 (35%)	
Unknown†	1 (2%)	4 (4%)	0 (0%)	
**Tumor cells with dense eosinophilic cytoplasm**				<0.0001
Negative	10 (19%)	71 (75%)	39 (78%)^‡^	
Focal	10 (19%)	16 (17%)	9 (18%)^‡^	
Positive (Diffuse)	32 (62%)	8 (8%)	2 (4%)^‡^	

^†^Cases in the unknown category are excluded from statistical analysis.

^‡^Out of a total of n = 50 wild-type cases with sufficient tumor for assessment of this feature.

Dense eosinophilic cytoplasm has been shown in previous studies of SBT to be a distinctive feature of tumor cells harbouring the *BRAF*-mutation [9, 10]. In the present study, we define such cells as having abundant dense/glassy eosinophilic cytoplasm occupying at least 50% of the cell area, and at least 2 times the amount of dense/glassy cytoplasm compared with non-eosinophilic cells in the tumor epithelium (Figure 1D-1F). Diffuse involvement, namely, the conspicuous presence of these cells at 10X objective in multiple fields of view, was found in 62% of SBTs with *BRAF^V600E^* mutation. In contrast, only 8% of *KRAS*-mutated and 4% of wildtype SBTs exhibited this feature (p < 0.0001, Table 1). The sensitivity and specificity of this morphologic feature in predicting *BRAF^V600E^* mutation was 62% and 93%, respectively. The focal presence of tumor cells with abundant eosinophilic cytoplasm did not have any discriminatory ability and was seen in close to 20% of SBTs, irrespective of the underlying gene mutation (Table 1).

**Figure 2:**
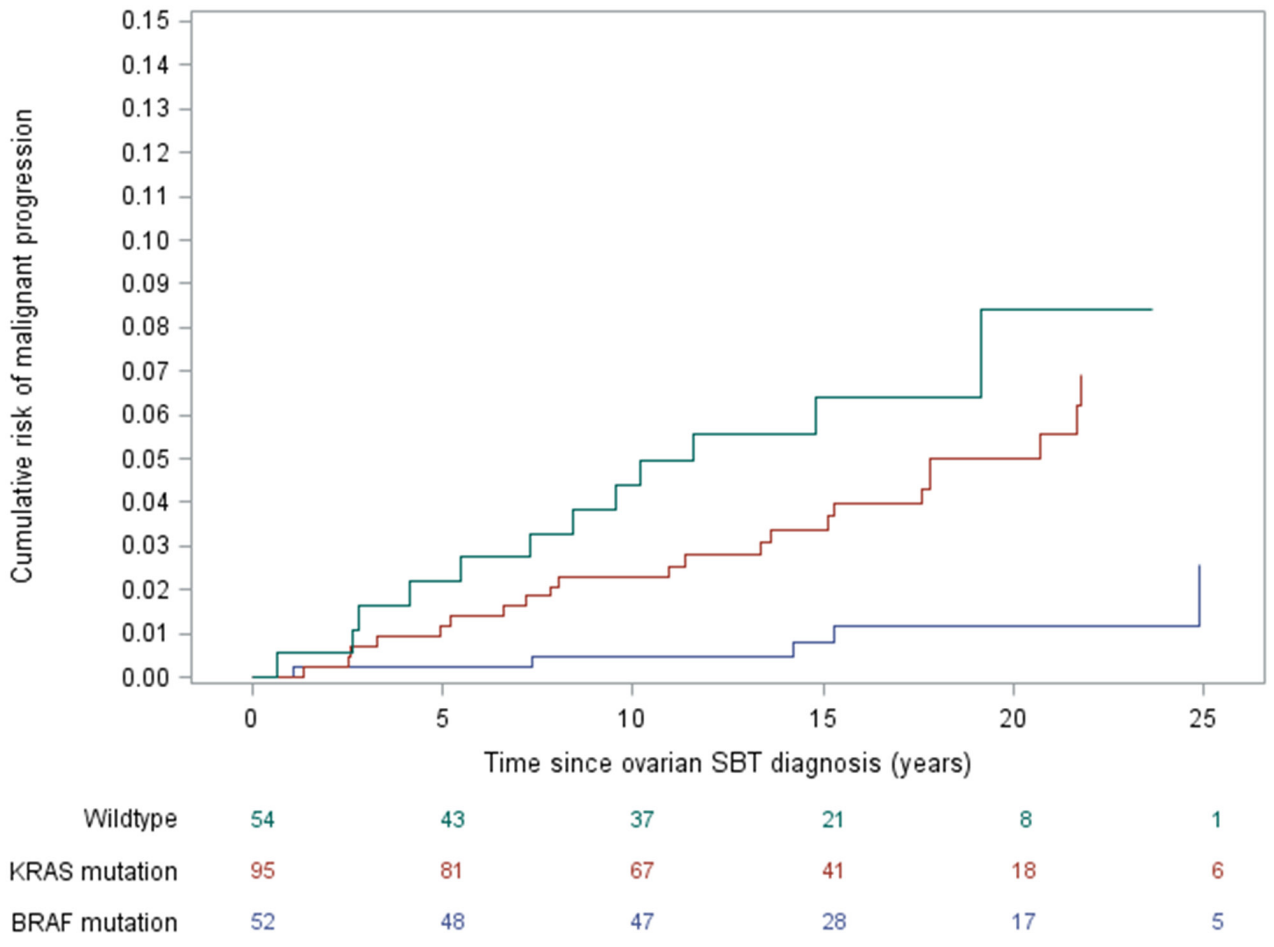
Risk of developing serous carcinoma over time, stratified by serous borderline tumor gene mutation status

The estimated cumulative risk of developing serous carcinoma is lowest in the *BRAF*-mutated group, with the 10-year risk being 0.5% compared to 4.4% for wildtype and 2.3% for *KRAS*-mutated SBTs (Figure 2). After adjusting for age and stage, compared to wildtype SBTs, the risk of subsequent serous carcinoma remains significantly lower among women with *BRAF*-mutated SBTs [HR 0.27 (0.08 – 0.93), p = 0.038] (Table 2). Additional adjustment for type of implant yielded similar results.

**Table 2:** Estimated risk of subsequent serous carcinoma by serous borderline tumor gene mutation

Gene mutation	Total number of women	Number of women with subsequent serous carcinoma	Estimated median time to progression† (years)	HR (95% CI)^*^	p-value
Wildtype	54	12	9.2	1.00	-
BRAF	52	22	19.7	0.27 (0.08 – 0.93)	0.038
KRAS	95	5	14.4	1.00 (0.45 – 2.23)	0.99

^†^time to progression derived using the Aalen-Johansen estimator.

^*^adjusted for age and stage (i.e. presence/absence of implants).

Subgroup analyses stratified by stage and SBT type showed similar trends. Considering Stage I cases only (n = 80), the hazard ratios for subsequent serous carcinoma, relative to wildtype SBTs, were 0.15 (CI 0.02 – 1.11) for *BRAF*-mutated SBTs and 1.19 (CI 0.22 – 6.45) for *KRAS*-mutated SBTs; considering Stage >I cases only (n = 120), the hazard ratios were 0.52 (CI 0.13 – 2.19) for *BRAF*-mutated SBTs and 0.79 (CI 0.28 – 2.21) for *KRAS*-mutated SBTs. Restricting the analysis to only conventional SBTs (i.e. excluding micropapillary SBTs, n = 177), hazard ratios were 0.36 (0.09 – 1.38) for *BRAF*-mutated SBTs and 1.34 (CI 0.51 – 3.46) for *KRAS*-mutated SBTs.

## DISCUSSION

Prognostication for SBT is difficult due to a number of factors. This is a low-grade neoplasm, with an indolent clinical course, requiring long-term follow-up to discern outcomes, with the most critical endpoint being progression to invasive serous carcinoma. Further complicating matters is the fact that pathologic diagnosis and staging is not always straightforward, particularly for the classification of extraovarian disease, which hampers efforts to reliably assess the prognostic relevance of clinicopathologic risk factors. To overcome the shortcomings of early work in this field, our group has been involved in epidemiologic studies incorporating rigorous central pathology review of SBTs identified in nationwide population registries from Denmark, with comprehensive long-term follow-up data [1, 11]. This work has confirmed the importance of traditional clinicopathologic features, including tumor stage and implant type, for predicting risk for subsequent carcinoma. The present study demonstrates that mutational status of *BRAF* gene is strongly associated with the clinical behavior of SBT, independent of stage and implant type.

Our findings are consistent with work by Wong et al., who first demonstrated a disproportionately low frequency of BRAF mutations in ovarian LGSC [only 1 of 43 (2%) cases] [12]. Subsequent work from an independent group confirmed an association between the *BRAF^V600E^* mutation with early stage disease and improved overall survival in a mixed cohort of 75 tumors diagnosed as either SBT or LGSC [13]. In another study, *KRAS* mutations were detected in 10 of 23 advanced stage ovarian SBTs that recurred as LGSC, but *BRAF^V600E^* mutation was detected in only one case [3]. Our current study extends these observations in an independent cohort of SBTs and represents the first with complete longitudinal long-term follow-up, allowing for stratification of risk for subsequent carcinoma by SBT gene mutation. The most sensitive assay currently available, ddPCR, was used for genotyping, given the inherent DNA degradation associated with paraffin samples stored for up to 30 years, along with the high stromal content frequently encountered in SBT.

Consistent with prior studies, *BRAF* and *KRAS* mutations were mutually exclusive in the vast majority of SBTs [5, 6]. However, we observed the presence of subclonal *KRAS* mutations in 4 *BRAF*-mutated SBTs. In these unusual cases, it is unclear whether *BRAF* and *KRAS* mutations co-exist within the same tumor cells, implying tumor progression, versus a “collision” event, involving separate tumor populations arising from different cells of origin. While the high MAF of *BRAF* mutation suggests the former scenario, this goes against our understanding of the overlapping biological roles of these oncogenes, as well as functional studies showing the detrimental effects of activating both *BRAF* and *KRAS* in the same cell. Given the rarity of this phenomenon, its significance remains unclear.

The relatively lower frequency of endosalpingiosis in women with *BRAF*-mutated SBT is an interesting and novel observation. Coupled with the association between *BRAF* mutation and localized disease confined to the ovary (and conversely, that *KRAS*-mutant and wildtype SBTs have a higher frequency of endosalpingiosis and advanced stage cases), this supports the hypothesis that endosalpingiosis can independently give rise to extraovarian implants in some cases and may explain, in part, the favorable prognosis of SBTs harboring the *BRAF* mutation [14].

In previous work, we have described the presence of tumor cells with abundant dense eosinophilic cytoplasm to be a characteristic morphologic feature in *BRAF*-mutated SBTs, a finding that has subsequently been confirmed independently by another group [9, 10]. Despite affirming this highly significant association in the present study, we show that histomorphologic assessment is not particularly sensitive in screening for the *BRAF^V600E^* mutation given the subjectivity in recognizing these eosinophilic cells. Nevertheless, recognition of this (prognostically favorable) histologic feature is important, as it may mimic the micropapillary variant of SBT, which is, conversely, associated with aggressive disease. In both entities, there is prominent epithelial tufting, imparting a crowded, hypercellular appearance at low magnification (Figure 1). However, upon closer scrutiny, micropapillary SBT exhibit slender papillae that measure at least 5X greater in length than width and consist of a homogeneous population of tumor cells. In contrast, *BRAF* mutation-associated tumors often show short, blunt papillae associated with scattered single cells or small clusters of cells with rounded contours and abundant eosinophilic cytoplasm. These characteristic cells may be exfoliated from the surface or present within the tumor epithelium, admixed with tumor cells showing usual cytomorphology. Micropapillary SBTs also have slightly more nuclear atypia than conventional-type SBTs with eosinophilic tumor cells.

An important caveat is that the present cohort represents a subset of the Denmark SBT population cohort, with an enrichment of advanced stage cases and inclusion of almost all the SBTs associated with subsequent malignant relapse. This explains the higher relative frequency of *KRAS* mutation observed, and under-representation of *BRAF* mutations, which we accounted for using stratified sampling and inverse probability weighting.

In summary, this study identifies *BRAF^V600E^* mutation as a favorable prognostic biomarker in ovarian SBT, supporting the concept of molecular subclassification of this pathologic entity for determining risk of subsequent development of serous carcinoma. Given the considerable interobserver variability with respect to diagnosis and staging for this entity, the development of an objective molecular classifier fills an unmet clinical need.

## MATERIALS AND METHODS

### Study population and case selection

Details of the population-based study cohort have been previously reported [1, 11]. Briefly, all women with a pathologic diagnosis of SBT between 1978 and 2002 in Denmark were identified in the Danish Pathology Data Bank and/or Danish Cancer Registry. Diagnostic slides were retrieved from 1,487 cases and reviewed by 2 gynecologic pathologists (R.V. and R.J. Kurman) blinded to all clinical information. Diagnostic terminology and criteria have been described in our previous publication [11]. Centralized histologic review confirmed the diagnosis of SBT in 1,042 cases. Women with concurrent invasive carcinoma in the ovary (n = 17) were excluded. Of the remaining 1,025 cases, 42 (4%) were associated with a subsequent diagnosis of serous carcinoma [39 (93%) cases low-grade and 3 (7%) cases high-grade serous carcinoma], with a follow-up period of at least 11 years from the time of diagnosis of SBT, up to 36 years. No patients were lost to follow-up. Clinicopathologic and histologic features were assessed from review of H&E slides, relevant information from the surgical pathology report (i.e. related to laterality, staging, etc.), and clinical records.

Following approval from institutional review boards, archival tissue specimens were procured retrospectively from all women who developed subsequent serous carcinoma with available tissue (39 of 42, “cases”), and a stratified random sample of 162 women with SBT that did not progress (“controls”), applying different sampling probabilities in strata by implants, with an oversampling among women with implants [96 of 116 with implants (>Stage I) versus 66 of 867 without implants (Stage I)]. The intentional enrichment of women with advanced stage was to account for the fact that advanced stage disease is most concerning for poor prognosis.

### DNA extraction

Ovarian tumor tissue was manually microdissected from 10-micron-thick unstained sections in areas with >70% tumor cellularity identified on corresponding H&E slides. Laser-capture microdissection (LCM) was performed to enrich for lesional tissue on cases with low cellularity and that were found to have mutations in both *BRAF* and *KRAS*. Microdissected tissues were subjected to genomic DNA extraction using the QIAamp DNA FFPE Tissue Kit (Qiagen, Valencia) as per the manufacturer’s instructions.

### Mutational analysis

Genotyping was performed by Digital droplet PCR (ddPCR), using the BioRad QX200 system. The following validated ddPCR mutation assays were obtained from Bio-Rad (Hercules, CA): *BRAF* p. V600E c. 1799T>A (dHsaMDV2010027); *KRAS* G12/13 Mutation Screening Kit (cat#1863506); *KRAS* p. G12C c.34G>T (dHsaMDV2510584); *KRAS* p. G12V c. 35G>T (dHsaMDV2510592); *KRAS* p. G12D c.35G>A (dHsaMDV2510596); and *KRAS* p. G12A c. 35G>C (dHsaMDV2510586). All samples were subjected to mutation analysis using the *BRAF*-V600E assay and the *KRAS* G12/G13 Mutation Screening Kit, a multiplex assay which screens for 7 common mutations in codons 12 and 13. Samples were subjected to *KRAS* G12C, G12V, G12D, and G12A mutation-specific ddPCR assays for definitive genotyping if found to carry a KRAS mutation by multiplex ddPCR.

The ddPCR reaction was comprised of 2X ddPCR Supermix (no dUTP), 0.5 μL of Uracil-DNA Glycosylase (UDG) (New England BioLabs, Ipswich, MA, USA), 1μL primer/probe assay reagent, and sample, up to a total volume of 20 μL. Droplets were generated using the Droplet Generator with an eight-channel DG8 cartridge and cartridge holder. Droplets contained 70 μL of DG oil per well and 20 μL of fluorescent PCR reaction mixture and were transferred to a 96-well PCR plate, which was subsequently heat-sealed with foil. PCR amplification was performed with the following cycling conditions: initial incubation at 37°C for 30 min, then 10 min at 95°C, followed by denaturation for 30 s at 94°C, annealing for 60 s at 55°C for 40 cycles; and final incubation for 10 min at 98 °C, ending at 4°C. After amplification, the 96-well plate was placed into the Droplet Reader (Bio-Rad, Hercules, CA, USA). Data were analyzed using the QuantaSoft analysis software (Bio-Rad). The threshold for a positive mutation call was set at an allelic frequency of ≥1.0%.

### Assessment for *BRAF* mutation-associated histology

Of 201 SBTs with molecular data, 197 were deemed adequate for microscopic evaluation for the presence of *BRAF* mutation-associated histology (namely, the presence of tumor cells with abundant eosinophilic cytoplasm) [9]. Adequacy was arbitrarily defined as at least 1 slide of ovary with tumor present in multiple high-powered fields.

Tumor cell morphology was assessed for the presence of round cells, with abundant dense/glassy eosinophilic cytoplasm occupying at least 50% the cell area, and at least 2X the amount of dense/glassy cytoplasm compared with non-eosinophilic cells in the tumor epithelium. Such cells were located within epithelium lining papillae and as detached single cells and small cell clusters that have exfoliated from the surface. The extent of involvement by eosinophilic tumor cells was scored as present (diffuse), focal/equivocal, or absent. Diffuse involvement was qualitatively defined as the presence of these characteristic cells easily recognizable using the 10X objective in multiple fields of view. Scoring was performed by a gynecologic pathologist (M.H.C.) blinded to the molecular data.

### Statistical analysis

For group comparisons, frequency data were performed by χ^2^-test, and age distribution was analyzed by a non-parametric Kruskal-Wallis test. Analysis of risk of malignant progression was performed using a case-cohort approach, where all cases contribute person-time only at the time of failure and applying inverse probability weighting to account for different sampling fractions in strata according to presence/absence of implants. Women were followed from the time of SBT diagnosis until death, emigration, progression or end of study, whichever came first; and death was treated as a competing event. The risk of progression according to time since SBT diagnosis in groups by SBT genotype was estimated using a weighted Aalen-Johansen estimator. The association between SBT genotype and risk of subsequent carcinoma was estimated by a weighted Cox proportional hazards model with time since SBT diagnosis as the underlying time scale. The underlying hazard was stratified according to implant status to control for differences in progression rates and furthermore adjusted for age as a continuous covariate. Two-sided 95% confidence intervals (CI) for the hazard ratio (HR) were estimated based on Wald’s test of the Cox regression parameter on the log (HR) scale.

## Abbreviations

SBT: serous borderline tumor
ddPCR: digital droplet polymerase chain reaction
