# Correction: *BRAF^V600E^*-mutated ovarian serous borderline tumors are at relatively low risk for progression to serous carcinoma

**DOI:** 10.18632/oncotarget.28001

**Published:** 2021-06-22

**Authors:** M. Herman Chui, Susanne K. Kjaer, Kirsten Frederiksen, Charlotte G. Hannibal, Tian-Li Wang, Russell Vang, Ie-Ming Shih

**Affiliations:** ^1^ Department of Pathology, The Johns Hopkins University School of Medicine, Baltimore, MD, USA; ^2^ Department of Obstetrics & Gynecology, The Johns Hopkins University School of Medicine, Baltimore, MD, USA; ^3^ Unit of Virus, Lifestyle and Genes, Danish Cancer Society Research Center, Copenhagen, Denmark; ^4^ Gynecologic Clinic, Juliane Marie Centre, Copenhagen University Hospital, Copenhagen, Denmark; ^*^ These authors have contributed equally to this work

**This article has been corrected:** In Table 2, the numbers in column 2, rows 2 and 3, were accidentally switched. The corrected Table 2 is shown below. The authors declare that these corrections do not change the results or conclusions of this paper.

Original article: Oncotarget. 2019; 10:6870–6878. 6870-6878. https://doi.org/10.18632/oncotarget.27326

**Table 2:** Estimated risk of subsequent serous carcinoma by serous borderline tumor gene mutation

Gene mutation	Total number of women	Number of women with subsequent serous carcinoma	Estimated median time to progression^†^ (years)	HR (95% CI)^*^	p-value
Wildtype	54	12	9.2	1.00	-
KRAS	95	22	14.4	1.00 (0.45 – 2.23)	0.99
BRAF	52	5	19.7	0.27 (0.08 – 0.93)	0.038

^†^time to progression derived using the Aalen-Johansen estimator.

^*^adjusted for age and stage (i.e. presence/absence of implants).

